# Quality Control and Variability Assessment of an Eight-Herb Formulation for Hypertension Using Method Validation and Statistical Analysis

**DOI:** 10.3390/molecules24081520

**Published:** 2019-04-17

**Authors:** Mariam Jarouche, Harsha Suresh, Mitchell Low, Samiuela Lee, Cindy Xu, Cheang Khoo

**Affiliations:** 1 Herbal Analysis and Pharmacological Laboratories (HAPL), NICM, Western Sydney University, Campbelltown, NSW 2560, Australia; mjarouchee@gmail.com (M.J.); mitchell.low@westernsydney.edu.au (M.L.); 2 School of Medicine, Western Sydney University, Campbelltown, NSW 2560, Australia; 3 National Measurement Institute, North Ryde, NSW 2113, Australia; samiuelalee@gmail.com; 4 Wentworth Institute, Surry Hills, NSW 2010, Australia; Cindy@win.edu.au (C.X.); khoo2031@gmail.com (C.K.)

**Keywords:** Qi Ju Di Huang Wan, hypertension, herb MaRS, HPLC-MS, PCA

## Abstract

Background—The quality control (QC) for commercial herbal formulations is sparse due to a lack of well-developed HPLC-ESI-MS/MS methods. Objective—This study reports the quantification of nine selected analytes for a commercial eight-herb formulation known as Qi Ju Di Huang Wan (QJDHW) used to relieve hypertension. Methods—An HPLC-ESI/MS method for the quantitation of analytes selected using the Herbal Chemical Marker Ranking System (Herb MaRS) was developed. The Herb MaRS ranking system which takes into account bioavailability, bioactivity, and physiological action related to its intended use and the commercial availability of the standard. After a method optimization, seven analytes were found to be ideal for quantitation. Results—The target analytes were identified using an electrospray ionization-tandem MS molecular breakdown comparison between the herbal peak and the commercial standard. The quantitative aspect of analyte variability of eleven samples was studied using fold variation. The fold variation of selected analytes among eleven samples ranged from 1.5 to 28.9. The qualitative aspect of variability was studied using principal component analysis (PCA) and hierarchical cluster analysis (HCA). Conclusions—There is a great degree of chemical variability in herbal formulations which are due to raw material harvesting times, storage techniques, and plant subspecies variability. Highlights—Commercial QJDHW formulations need to be standardised using HPLC-ESI-MS/MS to ensure better product quality control (QC) and product efficacy for the consumer.

## 1. Introduction

There has been a significant increase in the use and consumption of complementary medicines from traditional herbal sources in recent years. Many of these medicines often have multiple herbal components, and each of these components have associated pharmacological claims. The active ingredients within each medicinal herb can differ due to different sources of production. It is important for these types of manufactured formulas to have a standardised procedure for quality control (QC) in order to ensure the efficacy of the product [1,2,3,4,5,6]. In recent years, the method validation of phyto-markers has been the industry standard for the quality control of medicinal herbal products. This often involves the quantitation of one or two analytes to assess the chemical variability of a commercial herbal product. This type of analysis is woefully insufficient when it is used for the QC of chemical formulations with multiple herbal products [7,8,9,10,11]. A more comprehensive and rigorous method is required. This study demonstrates how the QC of an eight-herb Traditional Chinese medicine (TCM) formulation known as Qi Ju Di Huang Wan (QJDHW) [12,13,14,15] used in the treatment of hypertension can be achieved using an analytical method validation, a principal component analysis (PCA), and a hierarchical cluster analysis (HCA). 

The target analyte for each herb in the QJDHW formulation was selected using the Herbal Chemical Marker Ranking System (Herb MaRS) based on bioactivity against hypertension [16]. The Herb MaRS ranking system was developed at the National Institute of Complementary Medicine (NICM) and assesses the bioactivity, physiological activity, and the bioavailability of each analyte present in any herb or herbal formulation. The chemical structures of the target analytes are shown in Table 1 and the herbal composition in the QJDHW formula is shown in Table 2. 

QJDHW is typically consumed as an aqueous alcohol extract and has been shown to have a significant effect in decreasing the concentration of angiotensin in plasma and myocardium, reducing the endothelin (ET) content and improving the kidney blood stream in the rat tail murine model for essential hypertension [17,18]. In 2010, a systematic review of randomized controlled trials on the effectiveness and safety of QJDHW for the treatment of essential hypertension (10 randomized trials involving 1024 patients) suggested that QJDHW, when combined with antihypertensive drugs, is more effective in lowering blood pressure in the treatment of essential hypertension than antihypertensive drugs alone [19]. There are currently no clinical trials that have reported severe adverse events related to QJDHW use.

Commercially available QJDHW was acquired from eleven different sources. An analytical method validation was performed using UPLC (Ultra-Performance liquid Chromatography, Waters Corporation, Milford, MA, USA) ESI-MS/MS (Electrospray ionisation mass spectrometry, Waters Corporation). The quantitative variability of the formulation was then assessed using the eleven different sources. The data obtained from the chromatographic spectra were then statistically analysed using PCA and HCA to assess the qualitative chemical variability of QJHDW. In this manner, the chemical variability of commercially available QJHDW can be understood, and further improvements in QC can be implemented. 

## 2. Methods

### 2.1. Instrumentation 

A Waters ACQUITY UPLC system (Waters Corporation) coupled to a Waters Xevo TQ MS triple quadrupole mass spectrometer fitted with a Z-Spray^TM^ source was used in the analytical method development. Electrospray ionisation ((+)/(−) ESI-MS/MS) scanning mode and argon collision gas was used to identify each analyte in the herb against a commercially purchased analytical standard. Chromatographic separation was achieved using an Acquity BEH C18 (150 mm × 2.10 mm, 1.7 μm packing) column. The injection volume was set at 3 µL, and the column heater temperature was set at 28 °C at the start of each run. The results of the analyses were processed using Waters MassLynx^TM^ version 4.1 (Waters Corporation). 

An Adam AFA-210LC analytical balance (Adam Equipment Co., Perth, WA, Australia) and a Sartorius SE-2 micro analytical balance (Sartorius Australia, Melbourne, VIC, Australia) were used to weigh the samples and standards. The Powersonic 420 ultrasonic bath (Thermoline Scientific, Sydney, NSW, Australia) and Beckmann GP centrifuge from Beckmann Coulter (Beckmann Coulter, Sydney, NSW, Australia) were used in the extraction of the analytes from the herbal formulation. The extraction solutions were then passed through a Millipore 0.22 μm centrifuge filter with a microporous membrane purchased from EMD Millipore (Millipore, Billerica, MA, USA). 

### 2.2. Reagents, Chemicals, and Samples

LC grade acetonitrile (Mallinckrodt Chemicals Ltd., Chesterfield, UK) and LR grade ethanol (95%), methanol, and formic acid (90%) (Biolab, Adelaide, SA, Australia) were purchased. The gases used in the method validation were ultrahigh purity grade air, argon, helium, hydrogen, and nitrogen (Coregas, Sydney, NSW, Australia). Purified water (>18 MΩ cm) was obtained from an Elga Purelab Prima and Purelab Ultra high purity water system (Biolab, Adelaide, SA, Australia).

The analytical standards alisol C (98.6%), alisol B (96.0%), catalpol (98.0%), rutin (98.0%), luteolin (98.0%), and diosgenin (97.0%) were primary grade and purchased from Sigma-Aldrich (Sigma-Aldrich, Australia). The analytical standards paeoniflorin (98.7%), pachymic acid (97.9%), and cornuside (98%) were secondary grade and purchased from Phytomarker (Phytomarker Ltd., Tianjin, China). The primary grade standards have purity and spectroscopic characterisations while the secondary grade standards have purity by LC-PDA (Photo diode-array detection) only. The calibration curves were prepared with a standard purity adjustment.

Eleven samples of the Qi Ju Di Huang Wan herbal formula was obtained from suppliers in the Australian marketplace. There were five suppliers who provided the eleven samples. Most of these batch samples were donated, and the commercial donors requested to remain anonymous. Sample A-III was used for the method validation. 

### 2.3. Sample Extraction and LC Mobile Phase Preparation

The dried aqueous extract in pill form of the herbal formulation was decapsulated and passed through a ≤200 μm sieve. Approximately 0.5 g of each sample was weighed into a 10 mL conical flask, 10 mL 70% *v*/*v* aqueous methanol was added, and the mixture was sonicated for 1 h. The sample was then centrifuged at 4000 rpm (3466× *g*) for 10 min to pellet out the insoluble excipient. The supernatant was then passed through a 0.2 μm polyvinylidene difluoride (PVDF) membrane filter into 2 mL autosampler vials with glass insert for LC-MS analysis. The resultant liquid was stored at 4 °C and discarded after 48 h because the peak area of the analytes decreased by ≥2% after this time. Mobile phase A (0.1% aqueous formic acid) was prepared by the addition of 900 mL of water to a 1000 mL volumetric flask followed by 1.1 mL formic acid before making up to volume with water. Mobile phase B was acetonitrile. The mobile phase program is shown in Table 3. The mobile phases were degassed by sonication for 5 min and filtered through a 0.45 μm PVDF membrane filter before use.

### 2.4. Preparation of Stock Calibration Solution Using Analytical Standards

Two mixed stock standard solutions were prepared. The first mixed standard solution consisted of alisol B, pachymic acid, alisol C, rutin, catalpol, luteolin, and diosgenin. The second solution contained cornuside and paeoniflorin. This was done since the concentrations of cornuside and paeoniflorin were higher than the other analytes, and they showed a better solubility in ethanol than in methanol.

Individual solutions of 1000 μg/mL alisol B, pachymic acid, alisol C, rutin, catalpol, luteolin, and diosgenin were prepared by weighing 5.0 mg of each standard into a 5 mL volumetric flask and adding 3 mL of methanol. The solutions were then sonicated for 5 min or until the solid had dissolved. The solutions were cooled to room temperature and made up to volume with methanol.

The first mixed standard stock solution containing 40 μg/mL alisol B, 40 μg/mL pachymic acid, 25 μg/mL alisol C, 150 μg/mL rutin, 20 μg/mL catalpol, 70 μg/mL luteolin, and 20 μg/mL diosgenin was prepared by adding 1.50, 0.70, 0.40, 0.40, 0.25, 0.20, and 0.20 mL of the respective individual standard solutions into a 5 mL volumetric flask and made up to volume with methanol. The solution was then diluted 20-fold to give an intermediate mixed standard solution. This was done by diluting 50 μL of the original mixed stock solution into 1000 μL with methanol. 

The second mixed standard stock solution containing 25,000 μg/mL cornuside, and 25,000 μg/mL paeoniflorin was prepared by weighing 125 mg of the respective standards into a 5 mL volumetric flask and adding 3 mL ethanol before sonication for 5 min. The solution was then cooled to room temperature and made up to volume with ethanol. The solution was diluted 40-fold to give an intermediate mixed standard calibration solution. This was prepared by diluting 25 μL of the mixed standard stock solution into 1000 μL 95% aqueous methanol. 

These intermediate mixed standards were diluted as shown in Table 4 to give the mixed working standard solutions used to construct the calibration curve.

### 2.5. Recovery Studies 

To determine the analyte extraction efficiency of the method, an accurate weight of approx. 0.5 g of each herbal sample was transferred into 10-mL volumetric flasks. Then, the two spiking stock solutions for all the analytes were added to each sample for the 50%, 100%, and 200% recovery levels. The concentrations of the mixed spiking solutions were such that, for the 100% spike level, the resultant peak area and height would double that of the unspiked sample. Seven replicates were carried out for each spike level to give a total of twenty-one samples for all three spike levels. The spiking solvent was evaporated overnight in a fume hood.

### 2.6. MS Conditions 

The MS source conditions were set as follows: Nitrogen was the desolvation gas (800 L/h heated to 350 °C) and argon as the collision induced dissociation gas (0.15 mL/min) gave rise to a collision cell pressure of 4.3 × 10^−6^ Bar. The scan time was 0.05 s. The extractor cone voltage was 2 V, and the cone gas flow was 20 L/h. The source temperature was 150 °C, the capillary voltage was –3.2 kV in the (+) ESI mode and 2.00 kV in the (−) ESI mode. Two MRM (Multiple Reaction Monitoring) transitions (or product *m*/*z*’s) were chosen for each target analyte, with the most abundant transition used as the quantifier and the other transition used as the qualifier. The ESI polarity, precursor, and product ions were monitored, and the argon collision voltages required to achieve the transitions and the dwell times used are summarised in Table 5. The quadrupoles Q1 and Q3 operated with a peak width of 3 AMU and a scan time of 2 s.

### 2.7. Chemometric Analysis

The qualitative variability of the targeted analytes in the herbal formulation was studied using principal component analysis (PCA) and hierarchical cluster analysis (HCA) [20,21]. The software used was R (v.2.14.2) for data processing and statistical analysis. The raw chromatographic data of each analyte present in each herbal sample was converted into eleven separate comma-separated value (CSV) files. The paeoniflorin peak at approx. 1.6 min was shifted to approx. 0.8 min, and the alisol C peak at 3.2 min was shifted to 2.8 min to prevent peak overlap. The LC-MS chromatographic profiles obtained were then meshed into a single R.data file and placed in a neat stack plot. 

The R.data file was then loaded and accessed using the R (v.2.14.2) “chemometrics” package written by Varmuza and Filzmoser developed for PCA analysis [22]. The graphics wrapper for the data was provided by the ChemoSpec package written by Hanson [23]. The baseline of the dataset was then corrected to reduce the influence of noise present in the samples. The data was then normalized to negate the small differences due to changes in the concentration during the preparation of seven replicates. Finally, the data was binned to compensate for the effect of narrow peaks having shifting retention times. The region of interest containing the relevant peaks between 0.5 min and 4.0 min was selected for the analysis. The two options available for PCA were either classical or robust. While the classical method characterised a good deal of variance in the data set due to “outliers”, the robust method downplayed this aspect and used median absolute deviation to study variance. Classical PCA was chosen since the outliers needed to contribute to the variance to better understand the underlying variability and the quality of an herbal product. The “Pareto” scaling option was chosen to explain the variance because it is a compromise between “noscaling” which weighs peaks according to size and the “autoscaling” option which weights all peaks equally.

The specific reason for this choice was due to the cornuside peak having a much larger peak despite having a similar concentration to alisol C and pachymic acid in the samples. Both the robust and classical ellipses were shown in the PCA plot and the robust ellipse was chosen to identify potential outliers since it provided more definitive grouping of the samples due to its use of median absolute deviation. HCA was then performed on the data to corroborate the variance present in the PCA plot. 

## 3. Results and Discussion

### 3.1. Chromatographic Data and Recoveries

**Figure 1:**
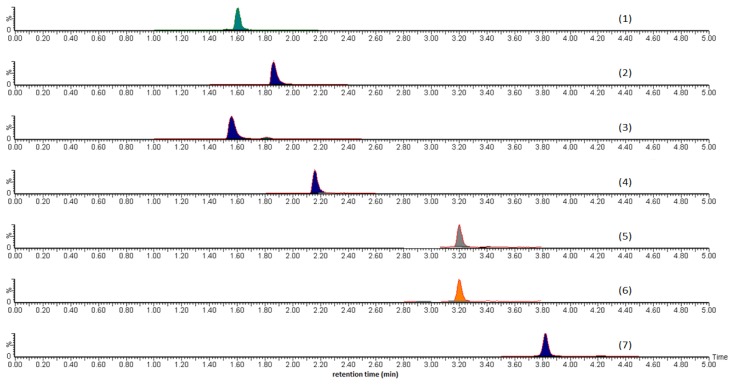
The representative MS chromatographic spectra of sample A-III containing (1) Rutin, (2) Cornuside, (3) Paeoniflorin, (4) Luteolin, (5) Alisol C, (6) Pachymic acid, and (7) Alisol B.

The representative chromatogram for the method validation sample A-III is shown in Figure 1. The monitored analytes show good recoveries in Table 6 ranging from 85% to 115% over three spiking levels. The 50% spiking level shows lower recoveries than the 100% and 200% spiking levels. This was due to a constant loss of the analytes caused by the staining of analyte to the glass volumetric flask used in the analyses. Ionisation suppression was not undertaken during the analyses since it would have caused lower recoveries for pure standard mixtures prepared in methanol and ethanol. If desired, an analyst using this method in the future could apply a recovery correction since the recovery RSDs (Relative standard deviation) are reasonably low. The instrumental SD (Standard deviation) was calculated by injecting the same sample multiple times (*n* = 7). This instrumental SD was then subtracted from the measured SD values before the RSDs were calculated for each analyte. During the analysis, catapol and diosgenin were not quantified or monitored further due to the presence of those analytes being below the minimum limit of quantitation (LOQ) and the limit of detection (LOD) in all eleven commercial samples. 

### 3.2. Peak Purity, MS Identity Confirmation, and Precision

The MS identity confirmation data is summarised in Table 7. The data is shown to be well within the tolerances described by the guidelines set out in the European Commission Directorate for Agricultural guidelines [24,25]. The analytes show a very high linearity, with *r^2^* > 0.99 for their calibration curves. Table 8 lists the data for the precision of quantitation. The analytical method was rigorously assessed for repeatability (intraday precision) and reproducibility (inter-day precision). The RSD deviation values shown in Table 8 employs the injection of seven replicates of the method validation sample A-III. The RSD measure for uncertainty was used instead of SD since it allows for an easier comparison between analytes in the same method with respect to a precision of quantitation. The RSDs reported in Table 8 show values for the inter-day precision of the method. The intraday precision was, on average, one-third that of the inter-day precision and is not shown. The stability (<2% degradation in analyte peak area) was between 72–96 h for all the analytes present in QJDHW and are shown in Table 8.

### 3.3. Analyte Concentrations and Fold Variation

The concentrations of the observed analytes across eleven samples are shown in Table 9. It is clear from the data that there is a great deal of quantitative variation characterised by the fold variation ranging from 28.9 for cornuside to 1.5 for Alisol B. The samples B-III and D-I are of especially poor quality since they are missing most of the analytes that were markers for the herbal components. Another curious observation was that luteolin was observed in high concentrations only in sample D-III. The quantitative variance observed could be due to growth conditions of the plant materials and poor batch standardisation, but the true reason becomes apparent when PCA is performed on the dataset. It is clear from the data that more stringent QC of commercial QJDHW is required in order to support the biological claims that are dependent on the presence of these analytes.

### 3.4. PCA and HCA

**Figure 2:**
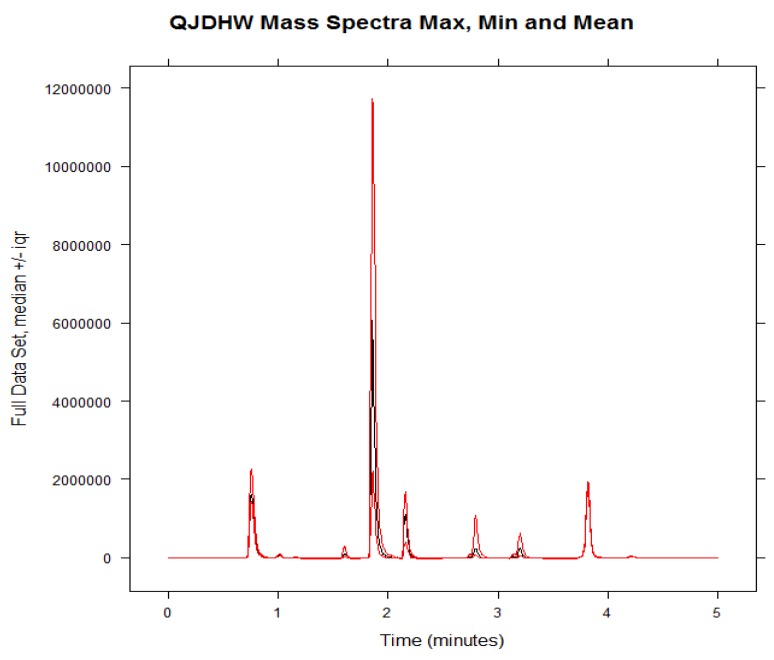
The min, max, and mean of the chromatographic data set before principal component analysis (PCA) was performed.

Traditionally, the variability of herbal formulations has been studied using quantitative methods alone. PCA was used to add a more qualitative dimension to the understanding of variability in commercial samples. When used in conjunction with qualitative data, it can afford a bigger picture into the variability present in a set of herbal samples. The min, max, and mean of the chromatographic data are shown in Figure 2 to demonstrate that the data has been properly assessed before PCA and HCA was carried out.

**Figure 3:**
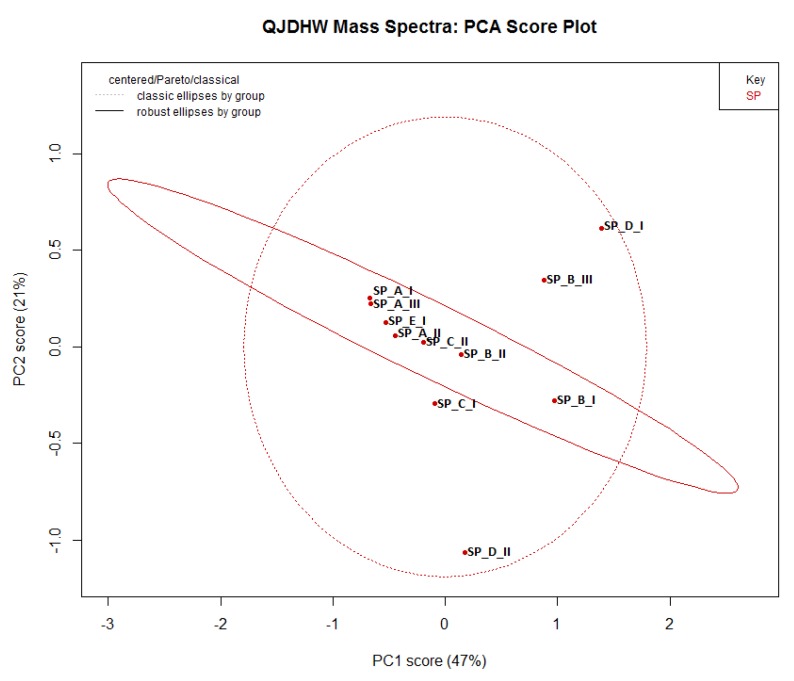
A PCA plot of the chromatographic data, where the SP prefix denotes “sample”’ followed by the label.

The PCA plot obtained is shown in Figure 3. The two principal components or PCs (PC1 + PC2) cumulatively explain 68% of the variance present in the sample, which is a very good score. Although more PCs can be used to explain the greater degrees of variability, two PCs were enough to explain most of the variability in this dataset. It was shown that samples D-I and B-III were clear outliers due to very low concentrations of the monitored analytes. D-II is also a clear outlier in a different direction due to the high concentration of luteolin present in the sample. All other samples group together very well and show some decent qualitative standardisation.

The difference of a single plant subspecies in an eight-herb formulation may not cause a great deal of variance, but two or three different subspecies of plant will cause a sample to highlight itself as an outlier. Sample D-II may be one such case due to its unusually high concentration of luteolin in comparison to the other samples. All the other samples show close grouping, and this suggests that the plant species labelled in the formulations are the same and the quantitative variance in the grouped samples was mostly due to different growth conditions and batch processing. 

**Figure 4:**
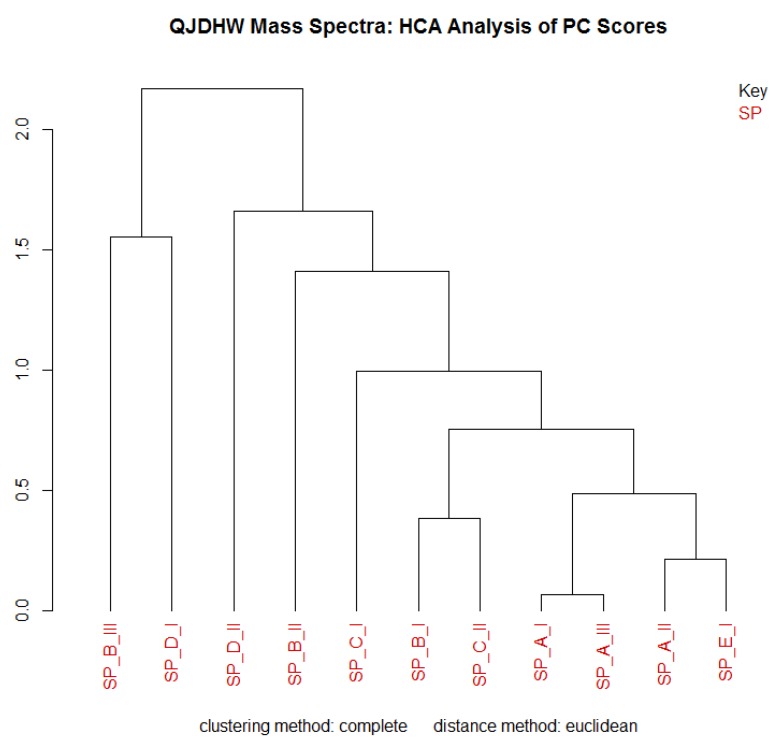
An hierarchical cluster analysis (HCA) plot of the chromatographic data, where the SP prefix denotes “sample” followed by the label.

HCA is a technique used to visualize the clustering of samples within a dataset. While PCA is useful to understand groupings in a sample set using its principal components, HCA allows us to show how closely or distantly related samples are using Euclidean distance and to establish a hierarchy of groupings. It is especially useful for a quick inspection in batch sample processing. Figure 4 illustrates that samples B-III and D-I were separated from the rest of the samples due to low concentrations of analyte. Sample D-II was also apart from the main grouping due to the possible presence of a different subspecies of *Chrysanthemum*. The HCA clustering largely corroborates the qualitative analysis of the samples using PCA.

## 4. Conclusions

A rapid 5 min UPLC-ESI-MS/MS method has been developed for the validation of the eight-herb formulation QJDHW using seven analytes. The quantitative variability of eleven different samples of the formulation was studied using fold-variation. The qualitative variability was studied using PCA and HCA. The samples show a great deal of chemical variability, and more standardisation and QC controls set out in this article are required in the future in order to support the pharmacological claims made by manufacturers.

## Figures and Tables

**Table 1:** The structures of the nine analytes monitored in Qi Ju Di Huang Wan (QJDHW).

Compound	Chemical Structure
Alisol B	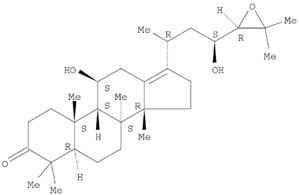
Pachymic acid	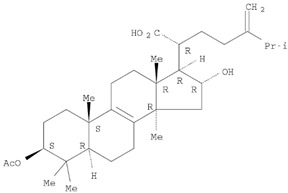
Alisol C	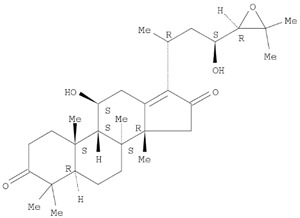
Rutin	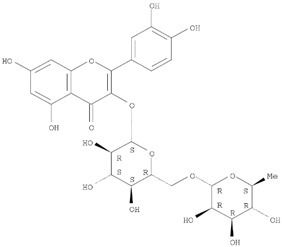
Luteolin	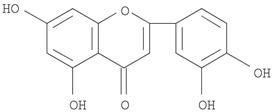
Cornuside	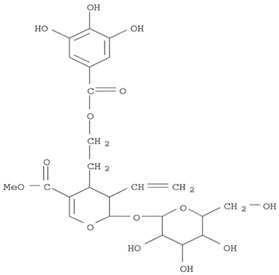
Paeoniflorin	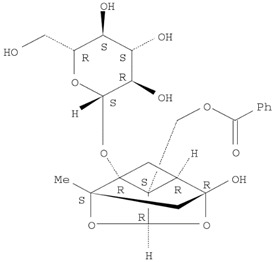
Catapol	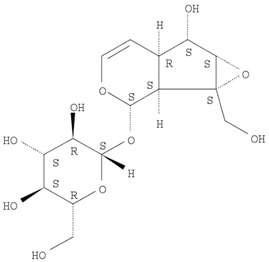
Diosgenin	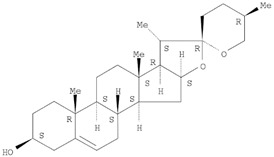

**Table 2:** The constituent herbs and analytes in QJDHW.

Pinyin Name	Botanical Name	% *w*/*w*	Analyte	Herb MaRS Ranking ^a, b^
Fu Ling	*Poria cocos* (Schw).	10.4	Pachymic acid	4
Gou Qi Zi	*Lycium barbarum* (L).	6.8	Rutin	5
Ju Hua	*Chrysanthemum x morifolium* (Ramat).	6.8	Luteolin	4
Mu Dan Pi	*Paeonia suffruticosa* (Andr).	10.4	Paeoniflorin	5
Shan Yao	*Dioscorea opposita* (Thunb).	13.8	Diosgenin	4
Shu Di Huang	*Rehmannia glutinosa* (Libosch).	27.6	Catapol	5
Shan Zhu Yu	*Cornus officinalis* (Siebold &Zucc).	13.8	Cornuside	4
Ze Xie	*Alisma plantago-aquatica subsp. orientale* (Sam).	10.4	Alisol B & C	4 & 4

^a^ Herbal Chemical Marker Ranking System [2]. ^b^ The ranking score ranges from 0 to 5, with 0 being the least and 5 being the most suitable.

**Table 3:** A mobile phase gradient program for the LC-MS/MS method.

Time (min)	% Water (with 0.1% *v*/*v* formic acid)	% Acetonitrile
Initial	90	10
1.0	75	25
1.5	55	45
2.5	35	65
3.0	25	75
3.5	20	80
4.0	0	100
4.5	90	10
5.0	90	10

Flow rate of 0.3 mL/min.

**Table 4:** The dilution volume for intermediate mixed standards.

Fold Dilution	Volume of Standard (µL)	Volume of Methanol (µL)
1/100	100	900
1/5	200	800
1/2.5	400	600
1/1.7	600	400
1/1.25	800	200
1	1000	0

**Table 5:** The UPLC-MS/MS monitoring conditions.

Analyte	ESI Polarity	Precursor *m*/*z*	Product *m/z*	Respective Cone Voltages (V)	Dwell Time (s)
Alisol B	+	[M + H]^+^ = 473	89, 121	26, 18	0.039
Pachymic acid	+	[M + H]^+^ = 529	295, 451	24, 18	0.039
Alisol C	+	[M + H]^+^ = 533	451, 469	20, 16	0.062
Rutin	−	[M − H]^−^ = 609	255, 271, 300	40, 62, 50	0.028
Catapol	+	[M + H]^+^ = 363	165, 183	12, 14	0.028
Luteolin	−	[M − H]^−^ = 285	133, 151	34, 26	0.028
Diosgenin	+	[M + H]^+^ = 415	253, 271	26, 18	0.039
Cornuside	−	[M − H]^−^ = 541	125, 169	20, 8	0.028
Paeoniflorin	−	[M − H]^−^ = 479	121, 449	54, 32	0.028

**Table 6:** The analyte recoveries.

Analyte ^c^	Spike Levels ^a^						Cumulative Results	
	50%		100%		200%			
	% Recovery	% RSD	% Recovery	% RSD	% Recovery	% RSD	% Average Recovery ^b^	% RSD
Pachymic acid	89.6	3.3	92.1	3.4	88.9	5.7	90.2	4.1
Alisol C	85.9	2.2	83.4	1.7	87.3	5.9	85.5	3.3
Luteolin	110.7	2.9	116.5	3.1	119.2	3.6	115.5	3.2
Paeoniflorin	84.8	3.4	92.3	2.9	83.0	5.4	86.7	3.9
Cornuside	116.8	2.3	108.3	3.2	101.1	3.2	108.7	2.9
Rutin	84.1	1.8	97.9	1.6	91.4	2.2	91.1	1.9
Alisol B	87.2	5.2	90.8	4.2	92.5	4.1	90.2	4.5

^a^ % Recovery ± % RSD calculated from seven replicate extractions and analyses. ^b^ Average recovery of all three spiking levels ± % RSD. ^c^ The limit of detection (LOD) was 0.005 mg/g and 0.003 mg/g for catapol and diosgenin, respectively. No recoveries were measured for these analytes.

**Table 7:** The identity confirmation of the analytes.

Analyte	Relative Intensity				Tolerances	
	*m*/*z*	Standard	Sample	Relative Difference (%) ^a^	Permitted Tolerance (%) ^b^	Pass/fail
Alisol B	121	100	100	-		
	89	65	66	1.5	±15	Pass
Pachymic acid	451	100	100	-		
	295	77	83	7.8	±15	Pass
Alisol C	469	100	100	-		
	451	45	46	2.2	±15	Pass
Rutin	300	100	100	-		
	271	65	66	1.5	±15	Pass
	255	34	35	2.8	±15	Pass
Luteolin	151	100	100	-		
	132	33	29	12	±15	Pass
Cornuside	169	100	100	-		
	125	41	40	2.4	±15	Pass
Paeoniflorin	449	100	100	-		
	121	75	72	4.0	±15	Pass

^a^ Relative difference = [(Intensity of sample – intensity of pure standard)/(intensity of pure standard)) × 100. ^b^ The maximum permitted tolerance of the European Commission Directorate for Agricultural guidelines is ±15 [7].

**Table 8:** The precision of quantitation.

Analyte ^d^	Linearity (*r^2^*)	Precision ^a^		LOD (mg/g) ^b^	LOQ (mg/g) ^c^	Stability (h)
		Amount (mg/g) ± % RSD	RT (min) ± % RSD			
Alisol B	0.9991	2.35 ± 4.21	3.81 ± 0.20	0.31	1.04	72
Pachymic acid	0.9993	3.99 ± 1.52	3.2 ± 0.01	0.18	0.62	72
Alisol C	0.9995	5.24 ± 0.76	3.2 ± 0.15	0.09	0.31	72
Rutin	0.9995	0.62 ± 4.80	1.6 ± 0.25	0.12	0.41	72
Luteolin	0.9996	0.59 ± 1.71	2.15 ± 0.23	0.04	0.14	72
Cornuside	0.9992	2.9 ± 1.64	1.86 ± 0.16	0.29	0.98	96
Paeoniflorin	0.9992	1.27 ± 3.15	1.5 ± 0.13	0.12	0.41	96

^a^ Average and RSD calculated from *n* = 7 replicates. ^b^ The limit of detection (LOD) is three times the standard deviation (SD) for each analyte in A-III. ^c^ The limit of quantitation (LOQ) is ten times the standard deviation (SD) for each analyte in A-III. ^d^ The limit of detection (LOD) was 0.005 mg/g and 0.003 mg/g for catapol and diosgenin, respectively. The concentrations of these analytes were not measured in the samples.

**Table 9:** The concentrations of target analytes.

Analyte	Concentration (mg/g) ± % RSD ^a^											Fold Variation ^b^
	A-I	A-II	A-III ^c^	B-I	B-II	B-III	C-I	C-II	D-I	D-II	E-I	
Alisol B	4.02 ± 3.22	2.88 ± 3.20	2.50 ± 4.31	2.50 ± 4.11	1.48 ± 5.11	2.88 ± 4.17	2.75 ± 3.37	2.88 ± 3.41	2.59 ± 3.64	2.37 ± 4.21	2.24 ± 3.39	1.5
Pachymic acid	3.85 ± 2.16	0.21 ± 7.83	4.05 ± 1.59	<LOD	1.16 ± 3.81	<LOD	0.38 ± 4.82	<LOD	<LOD	0.25 ± 6.59	0.70 ± 5.20	16.2
Alisol C	4.93 ± 3.50	0.68 ± 6.86	5.26 ± 1.29	<LOD	1.99 ± 3.24	<LOD	0.72 ± 4.74	0.25 ± 6.65	<LOD	0.42 ± 6.71	0.52 ± 6.11	12.5
Rutin	0.71 ± 7.12	0.98 ± 4.83	0.66 ± 4.10	0.7 ± 5.15	1.16 ± 4.18	<LOD	2.90 ± 3.13	1.82 ± 3.96	<LOD	0.66 ± 5.18	<LOD	4.4
Luteolin	0.53 ± 6.69	0.75 ± 5.89	0.58 ± 4.31	0.19 ± 5.82	0.27 ± 6.16	<LOD	1.22 ± 4.20	0.61 ± 5.33	<LOD	4.09 ± 5.27	0.88 ± 4.21	21.5
Cornuside	3.47 ± 1.37	1.23 ± 4.62	2.94 ± 2.49	0.52 ± 6.20	0.12 ± 7.22	<LOD	0.31 ± 5.83	0.90 ± 6.36	<LOD	0.24 ± 5.82	1.17 ± 4.84	28.9
Paeoniflorin	1.50 ± 2.91	1.10 ± 3.98	1.30 ± 3.91	0.26 ± 7.78	0.60 ± 6.22	0.70 ± 6.11	0.95 ± 4.63	0.74 ± 6.82	<LOD	0.95 ± 5.84	0.60 ± 6.23	5.7

^a^ Average calculated from seven replicates ± % RSD. ^b^ Fold variation = (highest concentration)/(lowest concentration), (<LOD values omitted from this calculation). ^c^ The analytical method validation performed on this sample.
